# A laboratory study of nonlinear changes in the directionality of extreme seas

**DOI:** 10.1098/rspa.2016.0290

**Published:** 2017-03-08

**Authors:** M. Latheef, C. Swan, J. Spinneken

**Affiliations:** Department of Civil and Environmental Engineering, Imperial College London, London SW7 2AZ, UK

**Keywords:** directionality, directional wave generation, extreme waves

## Abstract

This paper concerns the description of surface water waves, specifically nonlinear changes in the directionality. Supporting calculations are provided to establish the best method of directional wave generation, the preferred method of directional analysis and the inputs on which such a method should be based. These calculations show that a random directional method, in which the phasing, amplitude and direction of propagation of individual wave components are chosen randomly, has benefits in achieving the required ergodicity. In terms of analysis procedures, the extended maximum entropy principle, with inputs based upon vector quantities, produces the best description of directionality. With laboratory data describing the water surface elevation and the two horizontal velocity components at a single point, several steep sea states are considered. The results confirm that, as the steepness of a sea state increases, the overall directionality of the sea state reduces. More importantly, it is also shown that the largest waves become less spread or more unidirectional than the sea state as a whole. This provides an important link to earlier descriptions of deterministic wave groups produced by frequency focusing, helps to explain recent field observations and has important practical implications for the design of marine structures and vessels.

## 1. Introduction

In offshore and coastal engineering, the accurate description of extreme ocean waves is fundamental to the safe and efficient design of most marine structures, irrespective of whether they are fixed or floating. In considering such waves, it is well known that directionality plays a leading role in determining the temporal, spatial and spectral evolution arising in the vicinity of a large wave event. Taking this evolution as a whole, directionality will affect both the expected linear behaviour and the complex nonlinear wave interactions that characterize the largest and steepest waves. This paper is concerned with the latter, specifically nonlinear changes in the directional spread local to a large wave event. The motivation behind the work lies both in an improved understanding of the evolution of the largest waves in the most severe storms and in the accurate prediction of the fluid loads for design applications.

In considering this evolution, two types of laboratory data can be generated. The first relates to long random wave simulations that seek to provide the best possible representation of a real sea. The second concerns the generation of focused wave events. In this latter case, the amplitudes of the linear, freely propagating, wave components are assumed to be proportional to the underlying (target) wave spectrum, in accordance with the linear theory outlined in [1,2], and their phasing adjusted such that constructive influence or the superposition of wave crests arises at one point in space and time. In a linear sense, this superposition or focusing describes a deterministic wave event that is representative of the most probable shape of a large linear wave. In terms of the nonlinearity of the evolution, focused wave events are believed to be indicative of that which occurs in a random sea. However, there remain questions concerning the influence of the random phases and the fact that even the largest waves will not be perfectly focused.

Earlier work on the nonlinear amplification of crest heights highlights the importance of directionality. For example, previous studies [3,4] provide experimental observations and numerical calculations of focused wave groups. In both cases, the data indicate that while large increases in crest elevation beyond second order are observed in unidirectional waves, these rapidly reduce with increasing directional spread. Furthermore, the crest height statistics reported in [5,6] exhibit similar trends confirming that directionality has a similar, equally important, effect in random waves.

Numerical calculations of focused wave groups have also highlighted other nonlinear changes in the wave shape. These specifically relate to the aspect ratio, involving a compression in the mean wave direction and an expansion in the transverse or lateral direction. In effect, the waves become more long-crested, suggesting a local reduction in the directional spread. Evidence of this was first provided in [7] and further explored in [8]. Numerical calculations [9,10] also show that, with increases in the wave steepness, a directionally spread focused wave becomes more long-crested than predictions based upon linear dynamics. In considering these results, it is relevant to note that the second-order-bound waves produce no change in the crest length, confirming that these effects have their origins in the higher-order resonant interactions [8]. More recently, Adcock *et al*. [11] investigated the local changes in the directional spreading associated with large individual waves emerging from a random background. This work again highlights the nonlinear changes in directionality and is important in that it bridges the gap between the earlier work on focused waves and this study of random seas.

Further evidence supporting local nonlinear changes in the directional spread arise from the analysis of field data. For example, Monaldo [12] analysed space-borne synthetic aperture radar measurements and showed that the crests of the largest waves are longer (less directionally spread) than that predicted by an equivalent linear simulation. Similarly, Krogstad *et al*. [13] performed time-frequency analysis on data from the Ekofisk field in the North Sea and showed that the directional spread associated with the largest waves was smaller than the mean directional spread based upon the entire sea state. Alongside these findings, Krogstad [14] considered the influence of second-order wave spectra on heave/pitch/roll directional measurement and showed that the development of a frequency-dependent directional spread could be explained at frequencies below the spectral peak but not above. Building on this work Simanesew *et al*. [15] combined laboratory data, numerical calculations based upon the nonlinear Schrödinger equation and field observations to show that the static (*bound*) nonlinearity contributes to the low-frequency changes, whereas the dynamic (*resonant*) nonlinearity contributes to all frequencies; the former occurring rapidly and the latter taking some distance to evolve.

Although the development of frequency-dependent directional spreading is beyond doubt, a nonlinear increase in the overall directional spread is at odds with the accumulated evidence. The purpose of this paper is to examine the directionality of both steep seas and the largest waves arising therein; the latter determining whether local nonlinear changes in the directional spread are also observed in long random wave simulations. This adds to our understanding of nonlinear wave evolution, particularly the importance of directionality, addresses the applicability of calculations relating to focused wave groups and provides an explanation for the observed field data. Before undertaking this work, two additional ‘side’ issues need to be addressed. The first concerns the preferred method of generating directionally spread random seas in a laboratory wave basin; the second the optimum analysis procedure and the data to which it should be applied.

The paper continues in §2 with a brief review of earlier work, outlining the common measures of directional spreading. The generation of directionally spread random waves is addressed in §3, whereas §4 reviews the results arising from various directional analysis procedures and their dependence on the nature of the input data. Section 5 outlines the experimental methodology, together with details of the sea states considered. The main experimental results are presented in §6, with comparisons made to earlier numerical predictions. Finally, §7 offers some concluding remarks and highlights the practical implications arising.

## 2. Background

### (a) Directional spectrum

In characterizing a sea state, the directionality is usually combined with the frequency spectrum to describe a directional-frequency power spectrum

2.1 $$F(\omega ,\theta )=S_{\eta \eta }(\omega )D(\omega ,\theta ),$$

where *ω* and *θ* are the circular frequency and direction of propagation of a given wave component, *S*_*ηη*_(*ω*) is the frequency power spectrum and *D*(*ω*,*θ*) is the directional spreading function (DSF). In early work [16], *D*(*ω*,*θ*) was expressed as a Fourier series such that

2.2 $$D(\omega ,\theta )=\frac{1}{\pi }\left\{\frac{1}{2}+\sum_{n=1}^{\infty }[A_{n}(\omega )\cos n\theta +B_{n}(\omega )\sin n\theta ]\right\},$$

where

2.3 $$\begin{array}{rl} A_{n}(\omega ) & =\int_{-\pi }^{\pi }D(\omega ,\theta )\cos n\theta \;d\theta \\ \;\text{and}\;B_{n}(\omega ) & =\int_{-\pi }^{\pi }D(\omega ,\theta )\sin n\theta \;d\theta . \end{array}\}$$

Adopting this approach, key properties of *D*(*ω*,*θ*) are expressed in terms of the Fourier coefficients, *A*_*n*_(*ω*) and *B*_*n*_(*ω*). For example, the mean wave direction, *θ*_1_, is defined from the first pair of Fourier coefficients such that

2.4 $$\theta_{1}(\omega )=\arctan\left(\frac{A_{1}(\omega )}{B_{1}(\omega )}\right).$$

Likewise, the circular root mean square (RMS) spreading, *σ*_1_, is given by

2.5 $$\sigma_{1}(\omega )=\sqrt{2[1-\sqrt{A_{1}^{2}(\omega )+B_{1}^{2}(\omega )}]}.$$

Owing to the cumbersome nature of equation (2.2), *D*(*ω*,*θ*) is often expressed in alternative forms. One of the most widely applied expressions was introduced by Cartright [17] as

2.6 $$D(\omega ,\theta )=A\cos^{2s}\left(\frac{\theta -\theta_{m}}{2}\right),$$

where *θ*_m_ is the mean wave direction assumed to be constant across all frequency components (*θ*_m_ = *θ*_1_(*ω*), for all *ω*), *s* is the spreading parameter (commonly attributed to [18]) and *A* is a normalizing coefficient ensuring

2.7 $$\int_{\theta_{m}}^{\theta_{m}+2\pi }D(\omega ,\theta )\;d\theta =1;$$

the integral taken over all *θ* for any given value of *ω*. An alternative, and also commonly applied, distribution is given by

2.8 $$D(\omega ,\theta )=\frac{A}{\sigma_{\theta }\sqrt{2\pi }}\exp\left[-\frac{{\left(\theta -\theta_{m}\right)}^{2}}{2\sigma_{\theta }^{2}}\right],$$

where *θ*_m_ and *A* are as defined above and *σ*_*θ*_ is the standard deviation of the Gaussian distribution.

In considering these alternative distributions (equations (2.6) and (2.8)), *D*(*ω*,*θ*) is unimodal. As such, they are not directly relevant to bi-modal distributions in which there are distinct wind-sea and swell components, unless the two components are fitted independently and linearly summed. In contrast, the original form (equation (2.2)) makes no prior assumption about the shape, ensuring it can be entirely arbitrary. Adopting either of the alternative distributions (equations (2.6) and (2.8)) it can be shown that

2.9 $$\sigma_{\theta }=\sigma_{1}\;\text{and}\;s=\frac{2}{\sigma_{1}^{2}}-1.$$

Furthermore, there is no reason to assume that either *σ*_*θ*_ or *s* is frequency independent. Indeed, analysis of field data by, among others, [18–21] suggests that the directional spread is smallest at the spectral peak and largest in the tail of the distribution. However, the exact form of this frequency dependence remains the subject of ongoing work. To avoid any difficulties in this regard, all the sea states considered in this paper were generated using frequency-independent directional spreading.

### (b) Velocity reduction factor

In practical engineering calculations, directionality is often introduced via a velocity reduction factor, *F*_s_. This seeks to quantify the reduction in the predicted unidirectional velocities owing to the directional spread. This approach is commonly adopted because it allows the inclusion of directionality, albeit in an approximate form, without having to make fundamental changes to the methodology underpinning a typical unidirectional design calculation. In effect, convenience and familiarity are chosen over accuracy. In adopting this approach, *F*_s_ is defined by

2.10 $$F_{s}=\frac{\text{RMS in-line velocity in a directional sea}}{\text{RMS velocity in a unidirectional sea}}.$$

Alternatively, Tucker & Pitt [16] define the velocity reduction factor as

2.11 $$F_{s}={\left[\frac{{\bar{u}}_{c}^{2}}{\left({\bar{u}}_{c}^{2}+{\bar{v}}_{c}^{2}\right)}\right]}^{1/2},$$

where *u*,*v* describe the horizontal components of the water particle velocity in the (*x*,*y*) directions, *x* being aligned with the mean wave direction, and the subscript *c* denotes velocities arising beneath a wave crest. In this form, equation (2.11) can be applied either to a single wave event or to the sea state as a whole; the latter requiring an average to be taken across all wave crests (indicated by the overbar).

Adopting a linear representation and assuming *D*(*ω*,*θ*) is frequency independent, *F*_s_ corresponding to the directional distributions given in equations (2.6) and (2.8) can be approximated by

2.12 $$F_{s}={\left[\frac{s^{2}+s+1}{\left(s+1)(s+2\right)}\right]}^{1/2}\;\text{and}\;F_{s}={\left[\frac{1}{2}(1+\exp(-2\sigma_{\theta }^{2}))\right]}^{1/2}.$$

## 3. Directional wave generation

The generation of a target directional spread within the confines of a laboratory wave basin is a non-trivial issue. Previous work [22,23] was based upon a double summation method (DSM) in which all of the frequency components, *ω*_*i*_, were generated in all of the directions, *θ*_*j*_. In this case, the water surface elevation, *η*(*x*,*y*,*t*), is defined by the sum across all frequencies and directions,

3.1 $$\eta (x,y,t)=\sum_{i=1}^{N}\sum_{j=1}^{M}A_{ij}\cos[\omega_{i}t-k_{i}(x\cos\theta_{j}+y\sin\theta_{j})+\epsilon_{ij}],$$

where

3.2 $$\omega_{i}=i(2\pi \Delta f),$$

3.3 $$\theta_{j}=j\Delta \theta ,$$

and *ϵ*_*ij*_ is the starting phase for each (*ω*_*i*_,*θ*_*j*_) pair, chosen randomly from a uniform distribution lying in the range [0,2*π*]. In adopting this approach, Δ*f* and Δ*θ* correspond to the discretization of the wave spectrum in frequency and direction, *k*_*i*_ is the wavenumber of the *i*th frequency component and the amplitude *A*_*ij*_ is defined by

3.4 $$A_{ij}=\sqrt{2F(\omega_{i},\theta_{j})\Delta \omega \Delta \theta },$$

where *F*(*ω*,*θ*) is the directional frequency power spectrum defined in equation (2.1) and Δ*ω* = 2*π*Δ*f*. Within this representation, each frequency (i=1→N) is represented by *M* wave components, one travelling in each discretized direction (j=1→M). This gives a total of *MN* wave components.

When operating an array of wave paddles, there are practical limits (both large and small) to the size of the wave components that can be generated. This, in turn, limits the total number of wave components that can be employed in a given simulation. In this case, several authors, including Jefferys [24], have reported problems concerning the non-homogeneity of a sea state simulated using a DSM; the problem arises owing to the cancellation of wave components with identical frequencies but different directions. To overcome these difficulties, Miles & Funke [25] proposed what is commonly referred to as a single summation method (SSM). In this case, the frequency spectrum is discretized into bands of small but finite width, Δ*Ω*, within which successive components are generated in successive directions. As a result, any one frequency component travels in only one direction. Subject to this constraint, the amplitudes of the components within a band are defined such that they match the target directional spread, with *s* or *σ*_*θ*_ based upon the central frequency of that band. Furthermore, the amplitudes of the components in adjacent bands are scaled, so that the sum of the amplitudes across individual bands is consistent with the target frequency spectrum, *S*_*ηη*_(*ω*). Adopting this approach,

3.5 $$\eta (x,y,t)=\sum_{i=1}^{N}A_{i}\cos[\omega_{i}t-k_{i}(x\cos\theta_{i}+y\sin\theta_{i})+\epsilon_{i}],$$

with

3.6 $$\omega_{i}=\frac{i\Delta \Omega }{M},$$

where *M* again represents the total number of directions.

The approach employed in the present study is a variation of the SSM, referred to as the random directional method (RDM). In adopting this latter approach, any one frequency component is again generated in only one direction, hence the single summation, but the concept of dividing the frequency spectrum into small but finite bands in which successive components are generated in successive directions is abandoned. Instead, the direction of propagation of any one frequency component is chosen randomly, subject to a weighting function based upon the desired directional spread. In this study, the appropriate weighting is based upon a normal distribution with a standard deviation of *σ*_*θ*_ in accordance with the directional distribution outlined in equation (2.8). This method was previously adopted in [26,27] and formed the basis of the earlier laboratory study outlined in [6]. Given that this study is specifically concerned with the directionality of the sea state, the merits of these approaches need to be carefully considered.

**Figure 1.**
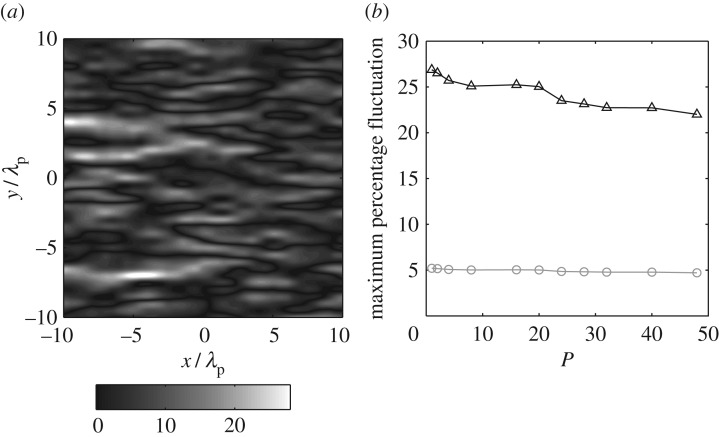
Linear calculations based upon the DSM. (*a*) The spatial variation of the mean wave energy calculated for a frequency resolution of Δ*f* = 1/1024 Hz; the data are presented as a percentage departure from the expected or target value. (*b*) The variation with *P* of the maximum percentage fluctuation in (black triangles) wave energy and (grey circles) *H*_s_.

It was noted above that several authors, including Forristall [23] and Jefferys [24], have indicated that a sea state generated in a laboratory wave basin using the DSM will not be ergodic. Evidence of this is provided in figure 1*a*. This shows the percentage deviation of the wave energy from the expected value, the data presented across a spatial domain of 20λ_p_ × 20λ_p_, where λ_p_ is the wavelength corresponding to the spectral peak. These data relate to a JONSWAP sea state with *H*_s_ = 0.15 m, *T*_p_ = 1.6 s, *σ*_*θ*_ = 30° and *d* = 1.25 m. This is equivalent to one of the laboratory test cases considered later and was simulated using linear random wave theory (LRWT) with a frequency resolution of Δ*f* = 1/1024 Hz. Figure 1*a* shows that large spatial variations in the wave energy arise, the maximum fluctuation being 27%. Furthermore, given the non-ergodic nature of the generated sea state, the significant wave height (*H*_s_) also showed significant variation in space; the maximum fluctuation being of order 5%. From the perspective of laboratory model testing, this variation is highly undesirable because it means that the severity of the sea state experienced by a model structure is dependent on the location of the model within the wave basin.

In considering this non-ergodicity, Jefferys [24] suggested that it would reduce with an increase in the frequency resolution. To confirm this, the sea state considered in figure 1*a* was regenerated with a frequency resolution of Δ*f*/*P*, where *P* was progressively increased. In all other respects, the calculations were identical; the spatial area held constant at 20λ_p_ × 20λ_p_. In each case, figure 1*b* describes the maximum departure from the expected wave energy (*E*_w_), expressed as |Δ*E*_w_|_max_/*E*_w_, and the maximum departure from the expected *H*_s_, again expressed as $|\Delta H_{s}{|}_{\max}/H_{s}$. Although both curves indicate a reduction in the spatial variability with increasing *P*, the rate of reduction is small, suggesting that *P* has to be very large (≫48) for the sea state to be considered ergodic. While this may be achievable in the practical operation of a laboratory wave basin, it is not desirable. The reasons for this are twofold. First, the generation of a very large number of infinitesimally small wave components presents difficulties in terms of the paddle transfer function; the latter linking the input signal to the actual waves generated. Second, the most representative sea states are generated using both random phases and random amplitudes. This is achieved by undertaking a large number of independent seeds (or realizations), each based upon a different set of random phases and amplitudes. As such, an appropriate random wave sample would incorporate 20 × 3 h simulations,^1^ rather than a single 60 h sample; the latter (corresponding to a large *P*) based upon one very large set of constant (deterministic) amplitudes. As an aside, it is also relevant to note that, in undertaking numerical simulations, calculations involving a very large number of wave components can lead to prohibitive computational costs, with few practical benefits. As a result, the present discussion concerning the preferred method of sea-state specification is equally appropriate to numerical calculations.

Neither the SSM nor the RDM suffers from non-ergodicity. As a result, the need to incorporate a prohibitively large number of frequency components is removed. Furthermore, both methods are relatively easy to implement, allowing a clear definition of both the frequency spectrum and the directions of propagation of the wave components. However, one difficulty that arises with the SSM concerns the balance between the number of frequency bands and the number of directions of propagation for a finite number of frequency components. This becomes more challenging when the directional spreading is increased, requiring more angles to be included in any one band. Given the removal of the finite bands in the RDM, difficulties of this type do not arise.

To evaluate the differences between the SSM and the RDM, the distributions of crest lengths associated with each of the two methods were compared. In making these comparisons, the crest length is a good measure of directionality; finite crest lengths being the first visual manifestation of directionality and easily calculated using LRWT. In the comparisons that follow linear calculations were performed based upon the (laboratory-scale) JONSWAP spectrum described previously: *H*_s_ = 0.15 m, *T*_p_ = 1.6 s and *d* = 1.25 m with a directional spread of *σ*_*θ*_ = 15° applied uniformly to all frequency components. In applying the alternative methods, 20 realizations of the sea state were made, each with a duration of 1024 s; the latter corresponding (approximately) to 3 h at full scale based upon the scaling discussed in §5. Using the surface elevation data generated at a single point, *η*(*t*) at *x* = *y* = 0, an up-crossing analysis was performed to identify individual wave crests. If *t*_c_ denotes the arrival time of each individual crest, the variation in the crest elevation in the transverse direction is defined by *η*(*y*) at *x* = 0 and *t* = *t*_c_. Adopting this profile, the crest length is defined as the distance between adjacent zero crossing points (*η*(*y*) = 0 on *x* = 0 and *t* = *t*_c_).

**Figure 2.**
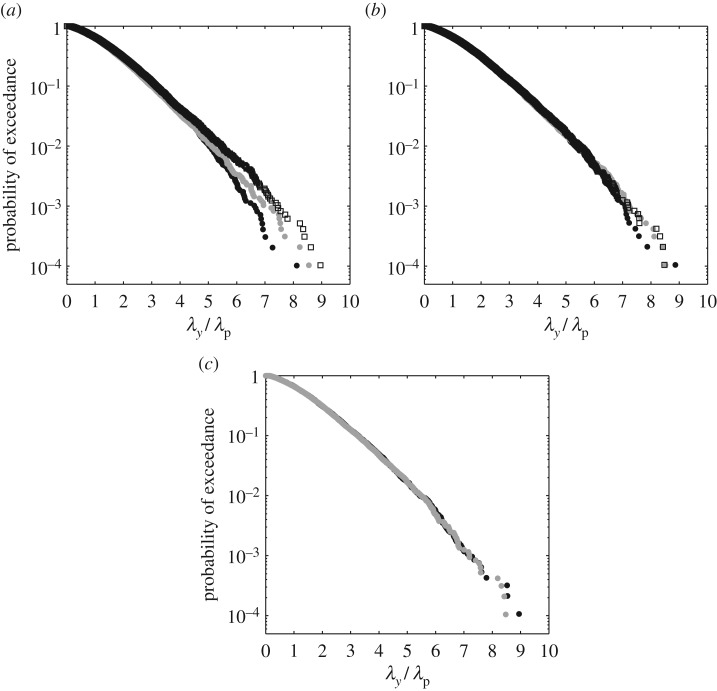
Exceedance probabilities for normalized crest length, λ_*y*_/λ_p_, based upon linear random wave calculations of a *σ*_*θ*_ = 15° sea state simulated using (*a*) the single summation method (SSM) and (*b*) the random directional method (RDM) for frequency resolutions (black solid dots) Δ*f* = 1/1024 Hz, (grey solid dots) Δ*f* = 1/2048 Hz and (black open squares) Δ*f* = 1/4096 Hz. (*c*) Comparison between data simulated using the (black circles) SSM and the (grey circles) RDM for frequency resolutions of Δ*f* = 1/8192 Hz and Δ*f* = 1/4096 Hz, respectively.

Figure 2 contrasts the exceedance probability of crest lengths arising in sea states generated using the SSM and the RDM. The purpose of this figure is twofold. First, to investigate the convergence of each method with varying frequency discretization and, second, to provide direct comparisons between the methods. Figure 2*a* concerns the SSM and figure 2*b* the RDM. In both cases, the crest lengths, λ_*y*_, have been normalized by the wavelength corresponding to the spectral peak period, λ_p_. In figure 2*a,* comparisons are made between three applications of the SSM with a frequency discretization of Δ*f* = 1/1024 Hz (*P* = 1), 1/2048 Hz (*P* = 2) and 1/4096 Hz (*P* = 4). In applying the SSM, the range of angles was held constant at −45°≤*θ*≤+45°. This corresponds to ±3*σ*_*θ*_, the applied truncation incorporating more than 99.6% of the wave energy defined by the full normal distribution. Further information concerning both the number of angles and the number of frequency bands, for each discretization, is given in table 1. Comparisons between the crest lengths presented in figure 2*a* confirm that a classical implementation of the SSM is very sensitive to the discretization of the frequency spectrum.

**Table 1.** Directional representation adopted in the single summation method (SSM).

frequency discretization, Δ*f*(*Hz*)	*P*	no. directional components	no. frequency bands
1/1024	1	25	64
1/2048	2	30	108
1/4096	4	40	161
1/8192	8	65	385

In contrast, figure 2*b* provides comparisons between a similar set of data generated using the RDM. Identical frequency discretizations are adopted and, in this case, the crest length distributions are shown to be less sensitive to the chosen Δ*f*. Indeed, ignoring the inherent variability that always exists in the tail of the distribution, the three RDM simulations define a converged distribution. Direct comparisons between the SSM and the RDM are presented in figure 2*c*. This concerns the crest length distributions arising in the same sea state; the SSM being based upon a frequency discretization of Δ*f* = 1/8192 Hz (*P* = 8) and the RDM on Δ*f* = 1/4096 Hz (or *P* = 4). The agreement between these distributions confirms that the directionality of the sea state can be generated using either method. However, a classical implementation of the SSM requires a finer discretization of the frequency range. Without this, the directionality will vary from the target or specified value.

As a final point, it should be noted that, in the theoretical limit of 1/Δf→∞, all three methods (the DSM, SSM and RDM) will give identical results. Indeed, it is only when seeking to limit the frequency resolution for practical wave generation reasons that data arising from the three methods of directional spreading can differ significantly.

## 4. Directional data analysis

Before performing any experimental measurements, a second set of linear calculations was undertaken to assess the preferred method of directional analysis and the input data on which it should be based. These calculations were again undertaken for a JONSWAP sea state with *H*_s_ = 0.15 m, *T*_p_ = 1.4 s and *σ*_*θ*_ = 20°.

In accordance with the discussion outlined in §3, 20 random realizations of the sea states were performed for all cases, each simulation being of 1500 s duration and all quantities sampled at 20 Hz. The computations were performed at six points corresponding to the centre and the five apexes of a pentagonal array of radius 0.5 m. The calculated quantities were the time-histories of the water surface elevation (*η*), the surface slopes (*η*_*x*_ and *η*_*y*_), the two horizontal components of the wave-induced velocity (*u* and *v*) and the corresponding vertical acceleration (*w*_*t*_); the velocities and accelerations being calculated at the centre point only. To avoid the gross errors associated with high-frequency contamination when using LRWT, the velocities and accelerations were calculated at an elevation of *z* = −0.2 m, where *z* is measured from the still-water level upwards. Within this section, the cross-spectral density function used within the directional analysis was estimated using the windowed Fourier transform techniques outlined in [21,28]; each signal being partitioned into segments of 1024 points (51.2 s in length), resulting in a frequency resolution of 0.0195 Hz.

Analysis of the linearly generated data involved three combinations of wave quantities. The first concerned the surface elevation time-histories, *η*(*t*), recorded at all six spatial locations, henceforth referred to as 6*η*. The second involved time-histories of the water surface elevation, *η*(*t*), and the two horizontal velocity components, *u*(*t*) and *v*(*t*), at the centre location. This is subsequently referred to as *ηuv*. The third combination was based upon the surface wave slopes, *η*_*x*_(*t*) and *η*_*y*_(*t*), and the vertical acceleration, *w*_*t*_, again determined at the centre location and referred to as *η*_*x*_*η*_*y*_*w*_*t*_. For each combination of inputs, calculations were based upon the maximum-likelihood method (MLM) following [29], the iterative maximum-likelihood method (IMLM) [30,31] and the extended maximum entropy principle (EMEP) [32]. Comparisons between these results showed that the best resolution of the directional spectrum was achieved using a combination of vector quantities and the EMEP. Evidence of this is given as follows.

**Figure 3.**
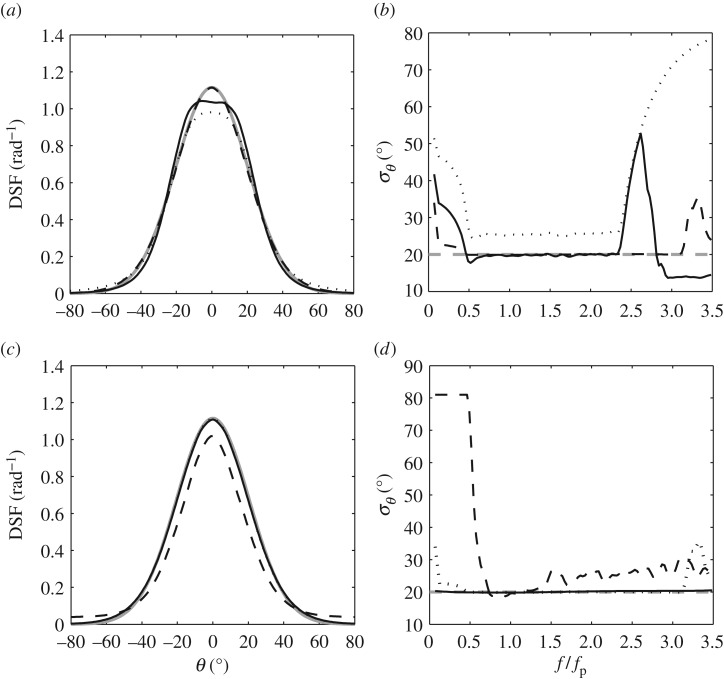
Directional wave properties based upon various linearly calculated input data and methods of analysis. (*a*) The directional spreading function (DSF) calculated using *ηuv* inputs to the (black dotted line) maximum-likelihood method (MLM), (black solid line) IMLM and (black dashed line) EMEP with comparisons to the (grey line) input conditions. (*b*) The corresponding *σ*_*θ*_ versus normalized frequency *f*/*f*_p_. (*c*) The DSF obtained for different input data (black dashed line, 6*η*; black dotted line, *ηuv*; and black solid line, *η*_*x*_*η*_*y*_*w*_*t*_) with comparisons to the (grey line) input conditions and (*d*) the corresponding *σ*_*θ*_ versus *f*/*f*_p_.

Figure 3*a*,*b* contrasts the results arising from the different methods of analysis; figure 3*a* describes the DSF averaged across the frequency range and figure 3*b* the variation of *σ*_*θ*_ with the normalized frequency, *f*/*f*_p_. All of these results are based upon the *ηuv* inputs (see below). Comparisons between these results confirm that the EMEP provides the best estimate of the directional properties, particularly in respect of the constant value of *σ*_*θ*_. Adopting the EMEP approach, figure 3*c*,*d* provides similar plots contrasting the three different combinations of input parameters. While the average shape of the DSF (figure 3*c*) is well defined in all but one case, the variation of *σ*_*θ*_ with *f*/*f*_p_ (figure 3*d*) is best resolved on the basis of vector quantities (*ηuv* or *η*_*x*_*η*_*y*_*w*_*t*_). In comparing these latter plots, it is important to note that the only differences arise for *f*<0.5*f*_p_ and *f*>3.0*f*_p_. Because there is little, if any, wave energy present within these ranges, the results are effectively identical. Moreover, because *η*(*t*),*u*(*t*) and *v*(*t*) are more easily measured in a laboratory environment, the *ηuv* input is the preferred option. In the sections that follow, the directional analysis is entirely based upon the EMEP with *ηuv* inputs.

## 5. Experimental procedure and test cases

Having established the best method of generating a directionally spread sea, the preferred method of analysis and the data on which this should be based, the primary aim was to identify any nonlinear changes in the directional spread, both for the sea state as a whole and for large individual wave events. To fully explore these effects, comparisons were required between sea states of varying steepness. In this regard, the long random wave records presented in [6] are highly relevant. Indeed, this study concerns a subset of four of these sea states, the details of which are given in table 2. The original data, full details of which are given in [6], concern time-histories of the water surface elevation, *η*(*t*), recorded over the working area of the wave basin. This study uses *η*(*t*) data recorded at one representative position, located 4.75 m downstream of the wave paddles. In addition, the velocity data, *u*(*t*) and *v*(*t*), outlined in §4 were recorded at the same spatial location and the analysis procedures described in §5 used to define the directional spread. In all cases, the random wave data, incorporating *η*(*t*), *u*(*t*) and *v*(*t*), were based upon 20 × 3 h simulations. Once again, the 3 h duration represents the equivalent ‘full-scale’ value based upon the scaling noted below and each simulation (or seed) involves a different set of random phases, amplitudes and directions; further details of the generated data are given in [6].

**Table 2.** Generated sea states, specified in terms of *H*_s_, *T*_p_ and *σ*_*θ*_.

sea state	*H*_s_ (m)	*T*_p_ (s)	*σ*_*θ*_ (°)	$\frac{1}{2}H_{s}k_{p}$
1	0.10	1.6	15	0.081
2	0.10	1.6	30	0.081
3	0.15	1.6	15	0.122
4	0.20	1.6	15	0.163

All of the laboratory data considered herein were generated in the 20 × 12 m wave basin located in the Hydrodynamics Laboratory within the Civil Engineering Department at Imperial College London, UK. Details of this facility are given in [6], with further information concerning the control and operation of the wave paddles provided in [33,34]. The sea states outlined in table 2 are based upon JONSWAP spectra, *S*_*ηη*_(*ω*), with a peak enhancement factor of *γ* = 2.5. In each case the DSF, *D*(*θ*), was based upon a unimodal Gaussian (equation (2.8)) specified in terms of *σ*_*θ*_. All of the observations (*η*(*t*),*u*(*t*) and *v*(*t*)) were undertaken in a constant water depth of *d* = 1.25 m, giving an effective water depth of *k*_p_*d* = 2.0, where *k*_p_ is the wavenumber corresponding to the spectral peak, calculated using the linear dispersion relation. Based upon these values, the sea states correspond to the deep end of the intermediate regime, with earlier work [6] having shown that the nonlinear interactions are typical of those arising in deep water. The sea-state parameters (*H*_s_ and *T*_p_), defined in table 2, represent the actual laboratory-scale values. However, it is important to put these parameters into context. If a length scale of *l*_s_ = 1:100 is applied, the corresponding time scale would be *t*_s_ = 1:10. Adopting these values: sea states 1 and 2 are representative of a 100 year design sea state in the southern North Sea, covering an appropriate range of directional spreads, and sea state 3 is representative of a 100 year condition in the northern North Sea or the Gulf of Mexico. In contrast, sea state 4 is very severe, being representative of a 10 000 year condition in a tropical cyclone. Based upon these arguments, it is clear that the four sea states outlined in table 2 not only cover an appropriate range of sea-state parameters (particularly sea-state steepness) but are also relevant to commonly applied design conditions.

In undertaking this study, the additional velocity data, describing the two horizontal velocity components *u*(*t*) and *v*(*t*), were recorded using a miniature acoustic Doppler velocimeter (ADV). This was mounted on a vertical steel rod of diameter *D* = 10 mm; the latter chosen to minimize any disturbance to the flow and, at the same time, to ensure that the system experienced no excitation (vibration) owing to the passage of the largest waves. The ADV was capable of measuring all three velocity components (*u*,*v*,*w*) within a sampling volume that is located 5 cm from the probe head. This volume is cylindrical in shape with a diameter of 6 mm and an adjustable length lying in the range 3–15 mm. With no requirement placed on the accuracy of the vertical velocities (*w*), the ADV was mounted so that the long axis of the sampling volume was vertical and its length maintained at *l* = 7 mm. Following the addition of artificial seeding, consisting of naturally buoyant hollow glass spheres with an average diameter of 10 μm, an average signal correlation of 99% was achieved in all tests. This enabled long time-histories of the two horizontal velocity components to be recorded with an estimated accuracy of ±3% and a typical data rate in excess of 100 Hz.

Although the addition of seeding material, local to the ADV, was essential to maintain both the accuracy and the sample rate, it can adversely affect the performance of any wave gauges. To avoid such difficulties the surface elevation records, *η*(*t*), and the kinematics data, *u*(*t*) and *v*(*t*), were recorded separately in independent or repeated runs. While this ensures both datasets are of optimal quality, it doubles the number of long random wave simulations that need to be undertaken and necessarily relies on the repeatability of the generated sea states. This latter point has been addressed in several recent studies, including [6]; the RMS errors between multiple repeated random wave records were less than 0.5% of the maximum crest elevation and close to the measurement accuracy of the wave gauges (±0.5 mm at laboratory scale).

Within the present tests, the ADV data were recorded at a fixed spatial location, 4.55 m downstream from the equilibrium position of the wave paddles. This corresponds to the position at which much of the surface elevation data reported in [6] were recorded and is sufficiently distant from the wave paddles to avoid any spurious evanescent wave modes. To apply the EMEP (§4), the data records must be continuous. While this implies no restrictions in respect of *η*(*t*), the two horizontal velocity components, *u*(*t*) and *v*(*t*), must be recorded beneath the level of the deepest wave trough. In practice, the large horizontal fluid velocities high in the water column ensured that this was the most difficult area to seed and, consequently, the most difficult area in which to maintain a consistently high data rate. Despite several attempts, these difficulties could only be overcome by moving the measurement location some distance beneath the deepest wave trough. Further discussion of this is given below.

## 6. Experimental results

### (a) Analysis of the overall sea-state characteristics

**Figure 4.**
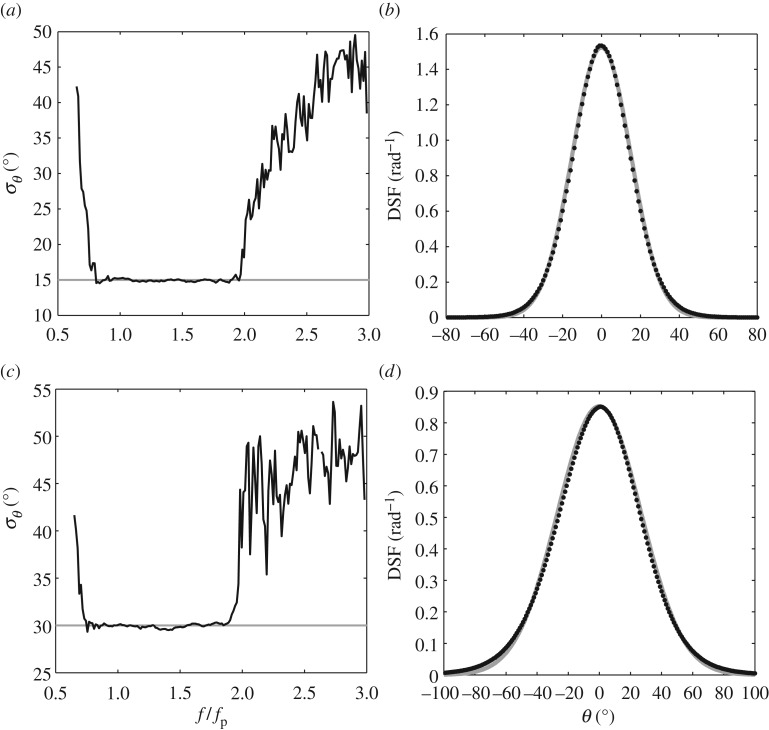
Directional properties of laboratory-generated sea states (*T*_p_ = 1.6 s, *H*_s_ = 0.10 m, $\frac{1}{2}H_{s}k_{p}=0.081$) based upon the EMEP with *ηuv* inputs; (*a*,*b*) relating to *σ*_*θ*_ = 15° and (*c*,*d*) to *σ*_*θ*_ = 30°. (black circles, black line) experimental data and (grey line) the input conditions.

Figure 4 concerns the directionality of sea states 1 and 2 in table 2. In both cases *H*_s_ = 0.10 m and *T*_p_ = 1.6 s, giving a sea-state steepness of 1/2*H*_s_*k*_p_ = 0.081. Based upon this steepness, both sea states are weakly nonlinear. Indeed, the only difference lies in the directional spread. This is applied uniformly to all frequency components, corresponding to *σ*_*θ*_ = 15° and 30°, respectively. Sea state 1 is considered in figure 4*a*,*b*; the first describes the calculated variation of *σ*_*θ*_ with non-dimensional frequency, *f*/*f*_p_, and the latter describes the average shape of the DSF, *D*(*θ*). The equivalent plots relating to sea state 2 (*σ*_*θ*_ = 30°) are presented in figure 4*c*,*d*. For all of the cases presented within this section, the EMEP has been employed as the preferred method of data analysis; the individual time-histories (*η*(*t*),*u*(*t*) and *v*(*t*) recorded at a single spatial location) were partitioned into segments of 6000 points, resulting in a frequency resolution of 0.0167 Hz in the directional-frequency spectrum. The kinematics data, representing a key input to the EMEP analysis, were recorded at *z* = −0.29 m. In accordance with the discussion given in §5, this was found to be the minimum elevation at which the ADV gave a continuous long record, without the occurrence of spikes or drop-outs.

**Figure 5.**
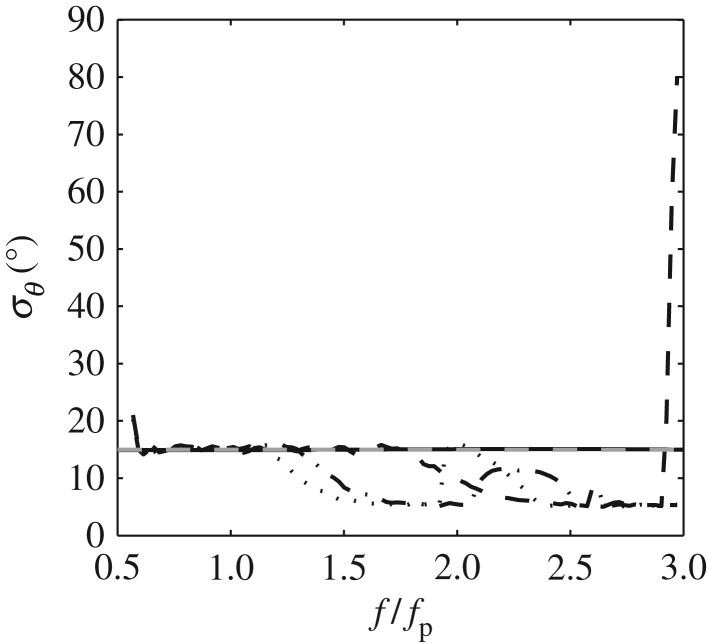
Comparative calculations based upon numerically generated data with *u*,*v* calculated at (black solid line) *z* = −0.2 m, (black dashed line) *z* = −0.35 m, (black dashed-dotted line) *z* = −0.65 m and (black dotted line) *z* = −0.8 m; the variation of *σ*_*θ*_ with *f*/*f*_p_ indicates the loss of resolution with depth. Numerical calculations based upon linear theory and (grey dashed line) the input condition.

The data presented in figure 4*a*,*c* confirm that the calculated values of *σ*_*θ*_ are exactly as generated, but only within a limited frequency range 0.85≤*f*/*f*_p_≤1.85. In considering this range, the lower limit simply reflects the absence of significant energy for *f*≤0.85*f*_p_. In contrast, the upper limit is at odds with the linear calculations presented in figure 3*a*,*c*; the latter plots suggesting that *σ*_*θ*_ should be maintained throughout the high-frequency tail. The explanation for this lies in the nature of the velocity data; specifically the vertical elevation at which it was recorded. This has been confirmed by comparisons with the results of an EMEP analysis based upon linearly predicted kinematics data. In these cases, the required kinematics data can be produced at any elevation beneath the level of the deepest wave trough, without the occurrence of spikes or drop-out. These results show that the upper limit of *f*/*f*_p_ for which a reliable estimate of *σ*_*θ*_ can be achieved is, in large part, determined by the elevation at which the kinematics data were recorded or predicted. Direct evidence of this dependence is provided in figure 5. This contrasts the results of the EMEP based upon kinematics data predicted at varying elevations, all other sea state parameters being identical to those in sea state 1 (table 2 and figure 4). In physical terms, the explanation for this dependence relates to the depth decay of the higher frequency wave components; the greater the depth at which the kinematics data are recorded (or predicted), the larger the proportionate reduction in the high-frequency energy. Clearly, there is a compromise to be found in which high-quality kinematics data are required, but they also need to be recorded as close as possible to the water surface.

**Figure 6.**
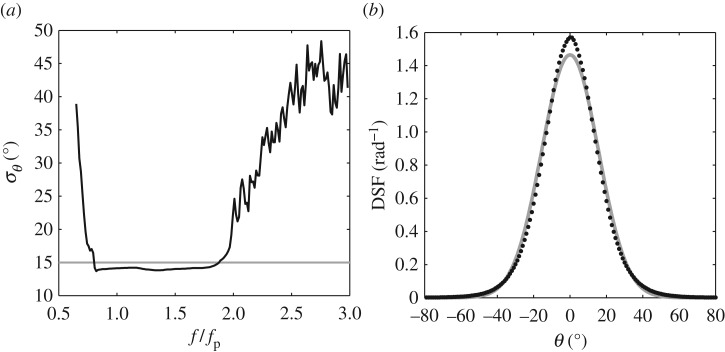
Directional properties of the laboratory-generated sea state (*H*_s_ = 0.15 m, *T*_p_ = 1.6 s, $\frac{1}{2}H_{s}k_{p}=0.122$, *σ*_*θ*_ = 15°); (*a*) *σ*_*θ*_ versus *f*/*f*_p_ and (*b*) directional spreading function (DSF). (black circles, black line) experimental data and (grey line) the input conditions.

Having established the effect of the depth decay, the *D*(*θ*) plots presented in figure 4*b*,*d* have been obtained by averaging over the frequency range appropriate to the correct *σ*_*θ*_ predictions. Subject to this limitation, it is clear that, in considering these two weakly nonlinear sea states (cases 1 and 2 in table 2), the calculated directional spreads presented in figure 4*a*–*d* are in close agreement with the generated values. Figure 6 provides the equivalent data, based upon the same analysis procedures, relating to sea state 3. This represents a steeper sea state defined by *H*_s_ = 0.15 m, *T*_p_ = 1.6 s (giving 1/2*H*_s_*k*_p_ = 0.122) and a frequency-independent directional spread of *σ*_*θ*_ = 15°. This sea state was previously considered in [6] and shown to exhibit significant nonlinear amplification of the crest heights. In this case, the increased steepness required the velocity data to be recorded at a slightly lower elevation, *z* = −0.34 m. Based upon the analysis of these data, *σ*_*θ*_ is again constant over the range 0.85≤*f*/*f*_p_≤1.85 (figure 6*a*). However, within this range *σ*_*θ*_≈14.3° and is therefore slightly below the generated value of *σ*_*θ*_ = 15°. This indicates that the sea state is, overall, less directionally spread (or more long-crested) than originally generated. Further evidence of this is given in figure 6*b*. This describes the average DSF, *D*(*θ*), which is clearly seen to be narrower (less directionally spread) than the target or input value.

**Figure 7.**
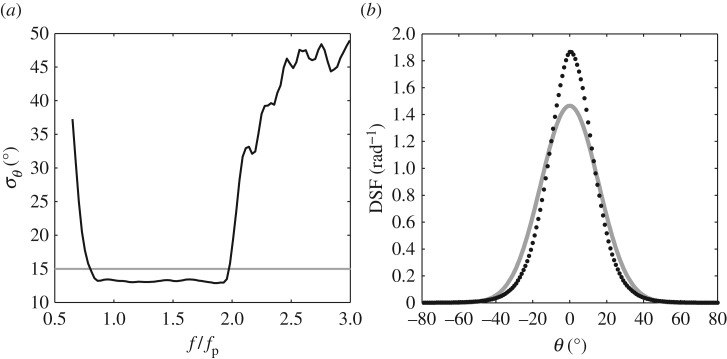
Directional properties of the laboratory-generated sea state (*H*_s_ = 0.20 m, *T*_p_ = 1.6 s, $\frac{1}{2}H_{s}k_{p}=0.163$, *σ*_*θ*_ = 15°); (*a*) *σ*_*θ*_ versus *f*/*f*_p_ and (*b*) directional spreading function (DSF). (black circles, black line) experimental data and (grey line) the input conditions.

Figure 7 provides a similar set of plots relating to the steepest sea state, case 4 in table 2. This is defined by *H*_s_ = 0.20 m, *T*_p_ = 1.6 m (giving 1/2*H*_s_*k*_p_ = 0.163) and *σ*_*θ*_ = 15°. With a further increase in the sea-state steepness, the kinematics data were again recorded at a slightly lower elevation, *z* = −0.39 m. Observations of this sea state, reported in [6], confirm not only that the crest heights exhibit significant amplification beyond second order, but also that the largest waves arising in the tail of the crest height distribution are subject to large-scale wave breaking. In this case, figure 7*a* confirms that the directional spread is again constant over the range 0.85≤*f*/*f*_p_≤1.85. However, this spread is characterized by *σ*_*θ*_≈13° compared with the input or generated value of 15°. Once again, this difference is highlighted by the comparison of the input (or generated) value and the calculated directional spread, *D*(*θ*), provided in figure 7*b*.

### (b) Analysis of individual wave events

To further investigate the nonlinear changes in directionality, a local (or wave-by-wave) analysis was also undertaken. With the EMEP based on frequency spectra appropriate to the sea state as a whole, this approach cannot be applied to individual wave events. However, with the availability of velocity data, the velocity reduction factor (*F*_s_) provides a convenient means of investigating the local directional spreading associated with individual wave events. While it is easy to apply equation (2.10) to a linearly calculated focused wave, it is less clear how it should be applied to laboratory data recorded in long random wave simulations. Specifically, two major difficulties arise:

(i) Calculating an *equivalent* unidirectional or in-line velocity time-history, *u*(*t*), for *σ*_*θ*_ = 0.
(ii) Relating the formula to short segments of the velocity time-history, rather than to the whole time series as is implied by equation (2.10).

Taking each of these points in turn, the first can be overcome using a simple (windowed) Fourier analysis. Recalling that the surface elevation, *η*(*t*), was recorded at the same spatial location as the velocity data, the phasing and the amplitudes of all the frequency components can be obtained by Fourier analysis. Assuming that all the wave components are travelling in the mean wave direction (*θ* = 0°), assigning the phase obtained from the Fourier transform and setting *x* = 0, an equivalent unidirectional velocity time-history can be obtained from LRWT; the accuracy of the predicted kinematics is further considered below.

To confirm the success of this approach, and to resolve the second issue noted above, linear random simulations of sea states with *H*_s_ = 0.15 m, *T*_p_ = 1.6 s and a variety of directional spreads (*σ*_*θ*_) were undertaken. To mirror the laboratory data recorded in sea states 1 and 2 (table 2), these linear simulations incorporated 20 seeds or simulations, each generated with different random phases and amplitudes. In addition to surface elevation time-histories, *η*(*t*), the two horizontal velocity components were generated at the same spatial location at an elevation of *z* = −0.34 m. Adopting these data, *F*_s_ was calculated using two methods, both based on equation (2.10). In the first approach, equation (2.10) was applied to the complete time-history, representing each random seed. In the second approach, an up-crossing analysis was performed on *η*(*t*), individual wave events identified and equation (2.10) applied to the individual segments of the velocity time-histories on a wave-by-wave basis. In the latter approach, an average *F*_s_, appropriate to the sea state as a whole, was obtained by taking the mean of the *F*_s_ values obtained for all individual wave events. In both approaches, the equivalent unidirectional velocity, *u*(*t*), was calculated using a Fourier analysis of the water surface elevation as outlined above.

**Figure 8.**
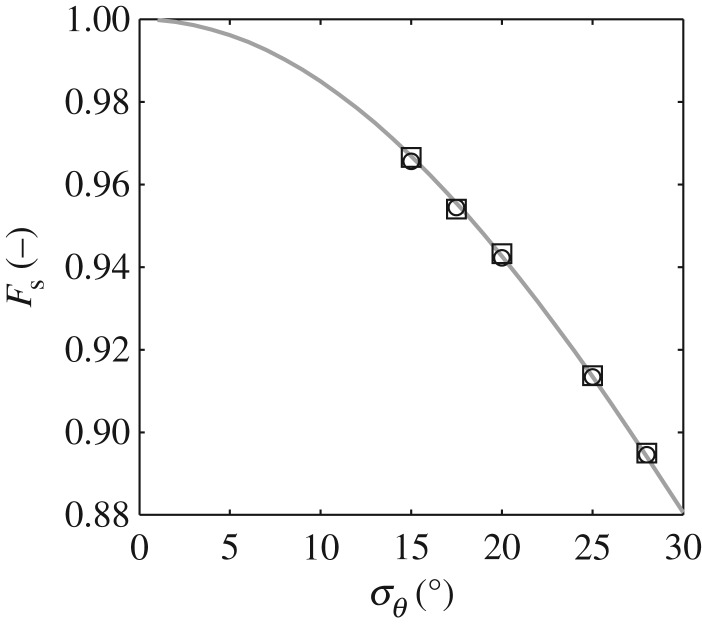
Directionality specified in terms of the velocity reduction factor (*F*_s_); calculations based upon the (solid grey) theoretical distribution and equation (2.10) (open circles) applied to the entire data record and (open squares) applied to individual waves and averaged over the complete record.

Figure 8 provides a comparison between the theoretical *F*_s_ calculated using equation (2.12) and the *F*_s_ calculated from the linearly simulated data employing equation (2.10). In the latter case, data relating to the two approaches outlined above are provided. Good agreement is obtained between all three methods. This goes some way to confirm the adopted procedures.

**Figure 9.**
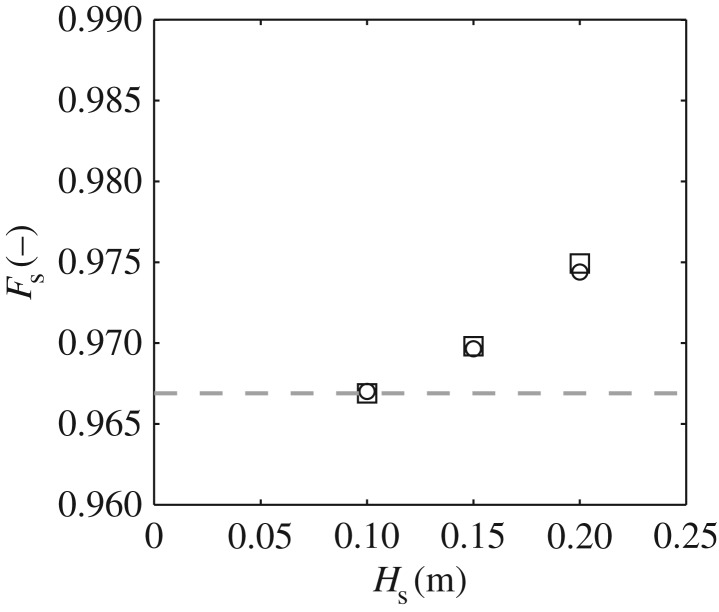
Variation in the sea-state averaged velocity reduction factor (*F*_s_) with the significant wave height (*H*_s_) for *σ*_*θ*_ = 15°; (grey dashed line) theoretical value based upon equation (2.12) with *σ*_*θ*_ = 15°, (open squares) revised estimate based upon equation (2.10) with *σ*_*θ*_ taken from figures 4, 6, and 7, (open circles) *F*_s_ based upon measured kinematics and averaged over individual wave events.

**Figure 10.**
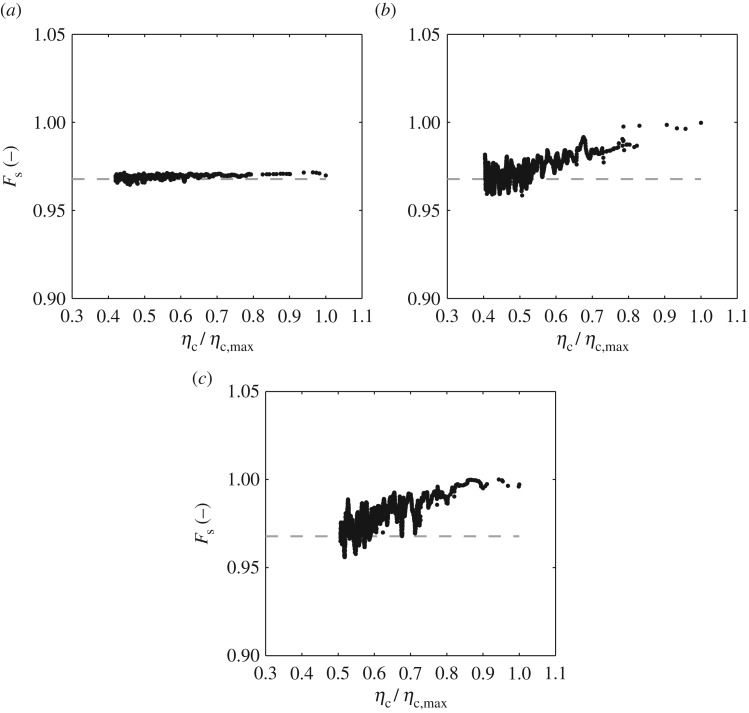
Velocity reduction factor (*F*_s_) for individual wave events ordered in terms of their crest elevation, *η*_c_/*η*_c,max_; based upon laboratory data recorded in a sea state with *T*_p_ = 1.6 s, *σ*_*θ*_ = 15° and (*a*) *H*_s_ = 0.10 m or $\frac{1}{2}H_{s}k_{p}=0.081$, (*b*) *H*_s_ = 0.15 m or $\frac{1}{2}H_{s}k_{p}=0.122$ and (*c*) *H*_s_ = 0.20 m or $\frac{1}{2}H_{s}k_{p}=0.163$.

Adopting the second method (noted above) for calculating the *F*_s_ of individual waves, figures 9 and 10 concern a re-analysis of the laboratory data outlined in table 2. All of the data relate to *T*_p_ = 1.6 s and *σ*_*θ*_ = 15°, with *H*_s_ = 0.10 m ($\frac{1}{2}H_{s}k_{p}=0.081$), 0.15 m ($\frac{1}{2}H_{s}k_{p}=0.122$) and 0.20 m ($\frac{1}{2}H_{s}k_{p}=0.163$); the specific cases having been considered earlier in figures 4, 6 and 7, respectively. Figure 9 concerns the mean or sea state averaged *F*_s_; the present values being denoted by the open circles and achieved by taking an average over all individual waves. These data are compared with *F*_s_ calculated from equation (2.12) based on the *σ*_*θ*_ values obtained from the directional analysis undertaken using the EMEP outlined in §6a; the individual results are presented in figures 4, 6 and 7 respectively. Data arising from this latter approach are presented in figure 9 using open squares. The agreement between these two approaches, once again, supports the appropriateness of the applied method. From a physical perspective, this alternative presentation of data shows that, with increasing sea-state steepness (or increasing *H*_s_ given a constant *T*_p_), the sea state as a whole becomes less directionally spread; the average or mean velocity reduction factor increasing towards 1.0.

Having further established the method of analysis, Figure 10 concerns the variation of *F*_s_ as a function of the normalized crest elevation (*η*_c_/*η*_c,max_). Figure 10*a* concerns the weakly nonlinear sea state defined by *H*_s_ = 0.10 m ($\frac{1}{2}H_{s}k_{p}=0.081$). In this case, there is (perhaps) a small reduction in the directional spread of the largest individual wave crests relative to the sea state as a whole; the latter are indicated by the horizontal dashed line. At first sight, this result appears at odds with the large isolated wave events investigated in [11]; the latter showing a reduced directional spread in waves of modest steepness. Given that the changes in directionality are due to third-order resonant (or near-resonant) effects [8], two possible explanations arise. First, this may be due to a difference in the effective water depth; the present results relating to *k*_p_*d* = 2.0 while the study by Adcock *et al*. [11] concerns deep water. Alternatively, the difference may be related to the effect of spectral bandwidth, with the study by Adcock *et al*. [11] being based on the nonlinear Schrodinger equation and therefore limited in terms of the effective bandwidth that can be accommodated.

In contrast, figure 10*b*,*c* provides similar plots for sea states with *H*_s_ = 0.15 m ($\frac{1}{2}H_{s}k_{p}=0.122$) and *H*_s_ = 0.20 m ($\frac{1}{2}H_{s}k_{p}=0.163$), respectively. Although the scatter in the calculated data for these two steeper sea states is larger than that in the weakly nonlinear sea state, two trends are clearly identified. First, the largest waves in these two sea states have a higher *F*_s_ when compared with the mean, suggesting that the largest waves are less directionally spread. Close examination of the data relating to the largest crests reveals that *F*_s_→1.0, but that *F*_s_≤1.0; the latter limit reflecting the fact that the introduction of even a small directional spread must produce a reduction in the wave-induced fluid velocities. The fact that the *F*_s_ values approach 1.0 in the largest wave cases suggests that these waves are close to unidirectional, at least as far as the fluid velocities high in the wave crest are concerned. Second, with a reduction in the crest elevation, *F*_s_ reduces back to the linearly predicted or sea-state averaged value. Contrasting the data presented in figure 10*b*,*c*, the number of wave events for which *F*_s_ approaches 1.0 is larger in the more nonlinear sea state.

The comparisons provided on figure 10 indicate that the conclusions derived from the directional analysis based upon the EMEP and presented in §6a (that sea states become less directionally spread with increasing steepness) can, in part, be explained by the fact that the largest waves in these sea states are becoming more unidirectional. Taken as a whole, the data presented in §6a,b, specifically figures 4–10, confirm that nonlinear changes in the directionality similar to those identified by [7–10] in relation to focused wave groups are equally appropriate to the largest waves arising in random seas.

## 7. Concluding remarks

This paper has reconsidered a subset of the sea states presented in [6]. The selected cases cover sea-state steepnesses (1/2*H*_s_*k*_p_) ranging from weakly to highly nonlinear, the latter incorporating significant wave breaking. With the addition of velocity data describing long time-histories of the two wave-induced horizontal velocity components, records of *η*(*t*), *u*(*t*) and *v*(*t*) at a single spatial location have allowed nonlinear changes in the directional spread to be investigated. Two approaches have been adopted. First, a conventional directional analysis, based upon the EMEP, has been undertaken to identify nonlinear changes in the directionality of the sea state as a whole. Second, a wave-by-wave analysis has been carried out with the velocity reduction factor used as a direct measure of the directionality. This latter approach has allowed the directionality associated with individual wave events within a random sea to be investigated.

The ‘conventional’ EMEP analysis has shown that, for weakly nonlinear sea states, the directional spreading derived from the laboratory data agrees well with the input conditions. This indicates that any nonlinear changes in directionality are small. However, as the sea state becomes steeper, the average directional spread is reduced, suggesting that the sea state becomes more unidirectional. As an additional point, the reduction in the directional spread remains frequency independent.

Similarly, the local wave-by-wave analysis showed that, with an increase in the steepness of the sea state, the largest waves are, on average, associated with a larger velocity reduction factor $\left(F_{s}\to 1.0\right)$. This indicates that the largest waves are more unidirectional than the sea state as a whole. With an increase in the sea-state steepness, the number of waves affected by this nonlinear change also increases. This, in turn, suggests that the overall change in directionality revealed by the ‘conventional’ EMEP analysis is due to the largest waves in the sea state becoming more unidirectional. Taken as a whole, the present results bridge the gap between earlier studies of deterministic focused waves [7–10] and recent field observations [12,13]; a key finding being that the local nonlinear changes in the directional spread observed in focused waves are also shown to occur in realistic, long random wave simulations.

## Footnotes

1 The 3 h duration being the equivalent ‘full-scale’ value, based upon the scaling discussed in §5.

## Data accessibility

The data on which the present paper are based can be made freely available on request to the corresponding author at c.swan@imperial.ac.uk.
